# Introducing GABLE: Lightweight infrastructure for longitudinal experiments using Google Apps Script

**DOI:** 10.3758/s13428-026-03205-8

**Published:** 2026-10-19

**Authors:** Ibrahim Berber, Ilke Kas, Tarun Sepuri, Brooke N. Macnamara, M. Cenk Çavuşoğlu, Amy L. Wilson-Delfosse, Elizabeth A. Krupinski, Philip J. Smith, Soumya Ray

**Affiliations:** 1 Department of Computer and Data Sciences, Case Western Reserve University, 10900 Euclid Avenue, Cleveland, OH 44106 USA; 2 Department of Electrical, Computer, and Systems Engineering, Case Western Reserve University, Cleveland, OH USA; 3 Department of Psychological Sciences, Purdue University, West Lafayette, IN USA; 4 College of Medicine, Northeast Ohio Medical University, Rootstown, OH USA; 5 Department of Radiology and Imaging Sciences, Emory University, Atlanta, GA USA; 6 Department of Integrated Systems Engineering, The Ohio State University, Columbus, OH USA

**Keywords:** Longitudinal studies, Study management, Participant communication, Experiment infrastructure

## Abstract

Longitudinal studies are essential for understanding how behaviors, skills, and psychological states evolve over time. While online platforms have made study development easier, researchers still face major logistical challenges when managing multi-session studies, especially when coordinating large numbers of participants, session timing, reminders, and compensation. In this paper, we introduce GABLE (Google Apps script-Based Longitudinal Experimentation), a lightweight infrastructure built using Google Apps Script. GABLE automates participant scheduling, e-mail communication, session tracking, and compensation. We demonstrate its effectiveness through two real-world psychological studies involving more than 850 participants with 20 sessions, and discuss its benefits, limitations, and potential extensions. GABLE addresses many challenges of running large online longitudinal studies while limiting the need for custom infrastructure or manual intervention.

## Introduction

Longitudinal studies are essential across a wide range of disciplines, including psychology (Prati & Mancini, 2021; Ramiz et al., 2021; Tang et al., 2021; Xiong et al., 2023), education (Szalay et al., 2021), and human–computer interaction (Gurcan et al., 2021; Melo et al., 2022; Skjuve et al., 2022), as they allow researchers to observe how individuals and systems change over extended periods of time. By capturing repeated measurements from the same subjects, longitudinal methods enable the study of temporal patterns that single-time-point or cross-sectional studies cannot reveal (Farrington, 1991). Example patterns include skill acquisition (Rowat et al., 2021; Vajsbaher et al., 2022; Wang et al., 2024), learning and memory consolidation (Kóbor et al., 2017), shifts in cognitive strategies (Esnaashari et al., 2023; Molnár & Kocsis, 2024; Zhou & Thompson, 2023), emotional adaptation (Valero-Moreno et al., 2021; Xu et al., 2024), and user behavior (Beeres et al., 2021; Rodrigues et al., 2022), as well as outcomes tracked in randomized controlled trials (Larsen et al., 2009). Longitudinal approaches are particularly valued for capturing real-world patterns of use and behavioral change often missed in traditional lab studies (Karapanos et al., 2012).

Recently, remote longitudinal studies have become increasingly viable thanks to widespread access to smartphones, wearables, and cloud-based platforms, as long as participants have the necessary devices and Internet access. These approaches reduce geographic and logistical barriers, enabling broader participation and more frequent, real-world data collection. Studies have shown that such designs can be feasible across diverse populations, including older adults and clinical patients, when paired with the right technology and participant support systems (Brewster et al., 2021; Creagh et al., 2022; Naz-McLean et al., 2022; Poirier et al., 2022). Many fully online behavioral and cognitive experiments require no special equipment beyond a web browser or mobile device. Indeed, many studies (e.g., Hampsey et al., 2022; Kochari, 2019) have demonstrated that web-based experiments can effectively replicate classic effects closely matching lab-based benchmarks.

Researchers who want to conduct web browser-based longitudinal studies typically face two major implementation challenges. The first involves developing and hosting the experiment itself, such as creating web-based tasks suited to test the study’s hypotheses and serving them to participants online. This may involve building the experiment within a graphical experiment-building platform (e.g., Qualtrics,^1^ Gorilla,^2^ E-Prime Go^3^) or coding it using a web-based framework or library (e.g., jsPsych, de Leeuw, 2015; lab.js, Henninger et al., 2022; PlanOut, Bakshy et al., 2014). Beyond building the task, researchers draw on a broader ecosystem of tools for hosting and delivery (e.g., JATOS, Lange et al., 2015; Cognition.run; Pavlovia/PsychoJS, Peirce et al., 2019), participant recruitment (e.g., Prolific, Palan & Schitter, 2018), and research with specific populations (e.g., Children Helping Science, Scott & Schulz, 2017).

The second challenge is managing participant flow over time, which is labor-intensive and error-prone when handled manually. This includes managing enrollment, scheduling, sending calendar invitations and reminder e-mails, tracking session completion, storing session scheduling information securely in a structured format, and distributing compensation, such as gift card codes. Because longitudinal studies depend on sustained engagement, persistent communication and personalized scheduling are essential for maintaining high participant retention (Cotter et al., 2002). This communication is often time-sensitive, with reminders and access links that may be tied to precise times that differ for each participant. For example, a session may be scheduled to open about 24 h after the previous one is completed, but within normal waking hours in the participant’s time zone. Addressing these needs at scale requires a system that not only automates logistics but also adapts to individual study schedules and participant timelines. For smaller studies with limited participants and fewer sessions, these tasks can often be handled manually. However, as studies scale, whether in sample size (e.g., hundreds of participants), number of sessions, or duration (e.g., weeks, months, years), such manual coordination becomes challenging and error-prone. Beyond the demand on researcher time, manual tracking and communication introduce risks such as missed or mistimed reminders that lock participants out of time-sensitive sessions, inconsistent handling that undermines protocol integrity, and errors in recording progress or delivering compensation, each of which can reduce retention and data quality. In these cases, a robust infrastructure that integrates various components (e.g., e-mail/calendar services, participant session management systems) becomes essential for ensuring smooth execution.

To address the logistical and coordination challenges of running multi-session studies, we developed GABLE (Google Apps script-Based Longitudinal Experimentation), a lightweight infrastructure built on Google Apps Script that automates participant management, reminders, scheduling, and compensation. Here, *lightweight* refers to computational and operational simplicity: Because GABLE runs entirely within the existing Google Workspace ecosystem, it does not require dedicated servers, installations, or external hosting. It can be deployed using either personal or institutional Google accounts. GABLE minimizes technical overhead and setup effort by leveraging built-in services such as Google Sheets, Gmail, and Google Drive, which are free, secure, and accessible. GABLE stores only study metadata (e.g., participant IDs, session completion status, scheduling information) in Google Sheets, while experimental data collected during tasks can be stored separately for security in any researcher-specified system (e.g., Qualtrics servers, Box, REDCap, OSF, institutional cloud storage).^4^ It integrates through configuration options with externally hosted experimental tasks and cloud storage systems, enabling researchers to run scalable, multi-session studies with minimal maintenance burden. These benefits, including reliable timing, consistent participant communication, and reduced errors, become increasingly relevant as studies scale in sample size, number of sessions, or duration–the conditions under which manual coordination becomes hardest to sustain. At the same time, because the initial setup is modest and reusable across studies, even smaller projects or researchers accustomed to a manual workflow can benefit from the same automation.

### Existing platforms

A variety of software platforms currently support aspects of longitudinal or multi-session research, each emphasizing different parts of the workflow, from task execution to participant management. Broadly, these systems can be grouped into (a) platforms for participant management and scheduling, and (b) platforms for experiment and task delivery.

#### Platforms for participant management and scheduling

Integrated Model for Patient Care and Clinical Trials (IMPACT; Weng et al., 2013) is a scheduling and participant-management platform designed for clinical research environments. It provides a real-time database of resources (exam rooms, equipment, staff) and enables study coordinators to schedule, track, and audit research visit appointments. IMPACT has been shown to reduce administrative burden and participant no-shows in clinical research (Butler et al., 2021), but such benefits remain specific to in-person, resource-managed settings rather than web-based behavioral studies. IMPACT does not support remote participation or online behavioral data collection. Recruitment, enrollment, longitudinal study setup, participant tracking, and payments are handled outside the system, and notifications and reminders are limited to visit-scheduling support.

Prolific (Palan & Schitter, 2018) is a participant-recruitment platform that enables researchers to access a large, vetted pool of online participants with rich prescreening and demographic filters. Recruitment and enrollment are streamlined: researchers select target criteria, set quotas or representative sample frames, and launch studies. Recently, Prolific introduced dedicated longitudinal projects (Haycraft Mee, 2026), which add automated multi-wave sequencing and scheduling, along with administrative tools such as retention and cost forecasting, retention tracking, and participant reminders. Communication tools include participant messaging and submission approval workflows. Incentive payments and rewards are managed in-platform. Prolific stores participants’ metadata on its servers and exports it in standard formats ideal for linking to external experiment platforms (such as Qualtrics); however, participant-management features (re-contact, long-term retention tracking) are less automated and depend on the researcher’s setup. The cost model is pay-per-participant plus platform fee, with academic discounts available.

Sona (Sona Systems, n.d.) is a participant-management and recruitment platform widely used in academic psychology departments. It supports posting studies, managing time slots, and coordinating both in-lab and online sessions (including up to four “multi-part” sessions for a single longitudinal study). Recruitment is managed through departmental subject pools, and scheduling is organized via predefined time slots within the system rather than flexible or calendar-based booking tools. Incentives are often managed via pre-specified credit values rather than integrated payment workflows. Participant metadata can be tracked through its researcher dashboard (e.g., participation status, no-shows), but Sona offers limited support for automatic session scheduling, progress tracking, and follow-up reminders.

#### Platforms for experiment and task delivery

Although our contribution focuses on automating participant management in large online longitudinal studies rather than automating task delivery, we provide a brief overview of task-delivery platforms for completeness. Readers interested in technical comparisons of task-delivery platforms can refer to reviews (Bridges et al., 2020). Broadly, these tools fall into two roles: *builders*, which are used to construct the experiment, and *hosts* (servers), which distribute it to participants and store the resulting data. Some platforms provide only one role, while others combine both.

jsPsych (de Leeuw, 2015) is an open-source JavaScript library for building and delivering browser-based behavioral experiments. It provides a modular, plugin-based structure that allows researchers to construct customized task timelines with precise control over stimuli, timing, and response collection. The library is well suited for a wide range of cognitive and perceptual paradigms and can be integrated into broader web development workflows. Recruitment, communication, and payment processes are therefore handled entirely outside the library.

OpenSesame (Mathôt et al., 2012) is a free, open-source experiment builder for the social sciences. Researchers assemble tasks through a graphical, drag-and-drop interface, with the option to add scripting for more advanced needs, making it suitable for a wide range of cognitive and perceptual paradigms. Its companion component, OSWeb, runs OpenSesame experiments directly in a participant’s browser, so a task built in the desktop application can be deployed online (Mathôt & March, 2022). Like other builders, OpenSesame/OSWeb focuses on task construction and delivery and is paired with a separate hosting server, typically a JATOS instance run on the researcher’s own or institutional server, to host the experiment and store data.

lab.js (Henninger et al., 2022) is a free, open-source experiment builder for online and in-lab studies. Researchers assemble a task through a visual, drag-and-drop interface, with the option to customize it in code for more advanced needs. Because lab.js builds but does not host the task, it exports to hosting services such as its companion, Open Lab (Shevchenko, 2022), which provides one-click deployment and data collection.

Gorilla (Anwyl-Irvine et al., 2020) combines both roles, building and hosting online behavioral experiments within a single platform. It offers a visual interface for constructing task flows and supports features such as conditional branching and random assignment to experimental conditions. Gorilla can accommodate multi-session designs by allowing researchers to make later tasks available after a delay or follow-up visit, though researchers must handle overall timing and longitudinal coordination outside the platform. Recruitment typically occurs through shareable links or external participant panels, and Gorilla logs standard task metadata such as completion status and timing. The platform stores all experiment data in its cloud environment and makes it accessible through built-in export tools.

Pavlovia (n.d.) is an online platform for hosting and delivering behavioral experiments built in PsychoPy and PsychoJS (Peirce et al., 2019). It enables tasks to run directly in participants’ browsers and supports a wide range of cognitive and perceptual paradigms, making it well suited for single-session or standalone experimental workflows. Multi-session studies are possible, but Pavlovia does not provide built-in mechanisms for scheduling follow-up sessions, automating return windows, coordinating longitudinal participation, recruitment, or payments.

JATOS (Lange et al., 2015) is a free, open-source server software for hosting online experiments. It distributes a study to participants as a shareable link and stores the resulting data, and is compatible with experiments’ built-in tools such as OpenSesame/OSWeb, lab.js, and jsPsych. Because JATOS is software rather than a ready-made service, researchers install it on their own or institutional servers; for those who prefer not to, MindProbe (https://mindprobe.eu) offers a free, public JATOS instance sponsored by the European Society for Cognitive Psychology. Participants are typically recruited on an external platform, redirected to the experiment on JATOS, and returned once finished. As a hosting layer, JATOS handles experiment delivery and data storage rather than participant scheduling, communication, or longitudinal coordination.

Cognition.run (https://www.cognition.run) is a free, web-based service for hosting online experiments built with jsPsych. Researchers write a task in its online editor, receive a shareable link for participants, and collect data that are stored and made available for download. Unlike self-hosted JATOS, it requires no installation, though it runs on proprietary software. Its role is limited to hosting and data collection, with recruitment and participant management handled elsewhere.

REDCap (REDCap Software, n.d.; Wright, 2016) is a browser-based data capture and research workflow tool widely used in clinical and academic settings. It supports the design of online surveys and databases with strong form logic (e.g., branching, calculated fields) and high customizability for user data rights and field validation (Harvey, 2018). Recruitment and enrollment must be managed externally: REDCap does not provide a built-in participant-panel or subject-pool system, and scheduling multi-session or longitudinal visits relies on its “events” and “arms” framework rather than a full calendar-booking interface. Communications such as survey invitations and alerts are supported (via built-in “Automated Survey Invitations”), but full longitudinal participant-management automation (e.g., automated follow-ups, time-based scheduling, limited grace-period options, or manual re-contact when required) is not provided within the platform.

#### Gaps in existing platforms

Across these platforms, no existing solution offers a unified, lightweight framework that automates all stages of longitudinal study management within a single, cohesive environment. Experiment builders and hosts such as Gorilla.sc, Pavlovia, jsPsych, OpenSesame/OSWeb, lab.js, JATOS, and Cognition.run excel at task presentation, delivery, and data collection but rely on external systems for participant tracking, scheduling, and follow-up. Clinical tools like IMPACT and REDCap provide robust infrastructures for appointment scheduling and data governance but are optimized for institutional, in-person trials rather than scalable, web-based behavioral research. Some participant-management systems automate longitudinal coordination. Prolific’s recent longitudinal projects automate multi-wave sequencing, scheduling, retention tracking, and participant reminders, while Sona supports subject-pool recruitment with basic multi-part scheduling. These capabilities, however, remain tied to each platform’s own recruitment pool and, for Prolific, a commercial fee-per-participant model. Below, we introduce GABLE, a system designed as an automation layer that coordinates all participant-facing processes around existing experiment platforms (see Table 1). It is built entirely on Google Workspace (Forms, Sheets, Drive, and Apps Script), which integrates recruitment, scheduling, communication, progress tracking, and incentive management into a single synchronized workflow. This flexibility enables GABLE to accommodate a wide range of longitudinal paradigms, from daily microtasks to multi-week training interventions, without requiring code modification. Unlike platform-bound solutions, GABLE is free for non-commercial use and open-source, works with any recruitment source and task host rather than a single participant pool, and exposes its scheduling, communication, and compensation logic for full customization. By coupling this accessibility with customizable configuration, GABLE bridges the gap between task-hosting systems and participant-management tools, providing a lightweight, scalable solution for online longitudinal research.

**Table 1:** Automation levels of key participant management features across other platforms and GABLE

Platform/framework	Recruitment & enrollment	Scheduling & session management	Communication & notifications	Incentive management	Participant data management	Administrative oversight	Access model
*Task builders & hosts*							
Gorilla.sc	P	✗	P	P	✓	P	Paid per participant
jsPsych/lab.js	–	–	✗	–	P	–	Free for commercial use
Pavlovia	✗	✗	–	–	P	P	Paid per participant
JATOS	✗	✗	✗	–	P	P	Free for commercial use
*Participant management & recruitment*							
IMPACT	✗	✓	✗	–	P	P	Institutional/site-based access
Prolific	✔	✔	P	✔	P	✓	Paid per participant
Sona Systems	✔	✔	✔	P	P	✓	Institutional license
REDCap	P	P	✔	–	✔	✓	Institutional license
Qualtrics	P	✗	P	P	P	P	Subscription/institutional license
GABLE	✓	✓	✓	✓	✓	✓	Free for non-commercial use

✓ = Highly automated; P = Partially-automated; ✗ = Not automated; — = Not implemented or not applicable

Rather than replacing task-delivery tools, GABLE is designed to interface with them. Because it coordinates participants around an externally hosted task rather than delivering the task itself, GABLE can work with a task built or hosted using any interface as long as the task records participant progress to a shared storage location that GABLE reads. This makes it compatible with a broad range of tools, from coded frameworks such as jsPsych or lab.js to custom web applications. We describe the integration mechanism in the following section and provide a worked example in the GABLE repository.^5^

The following section provides a detailed overview of GABLE’s architecture and operational workflow, illustrating how each component supports end-to-end participant management.

### Platform overview

**Fig. 1:**
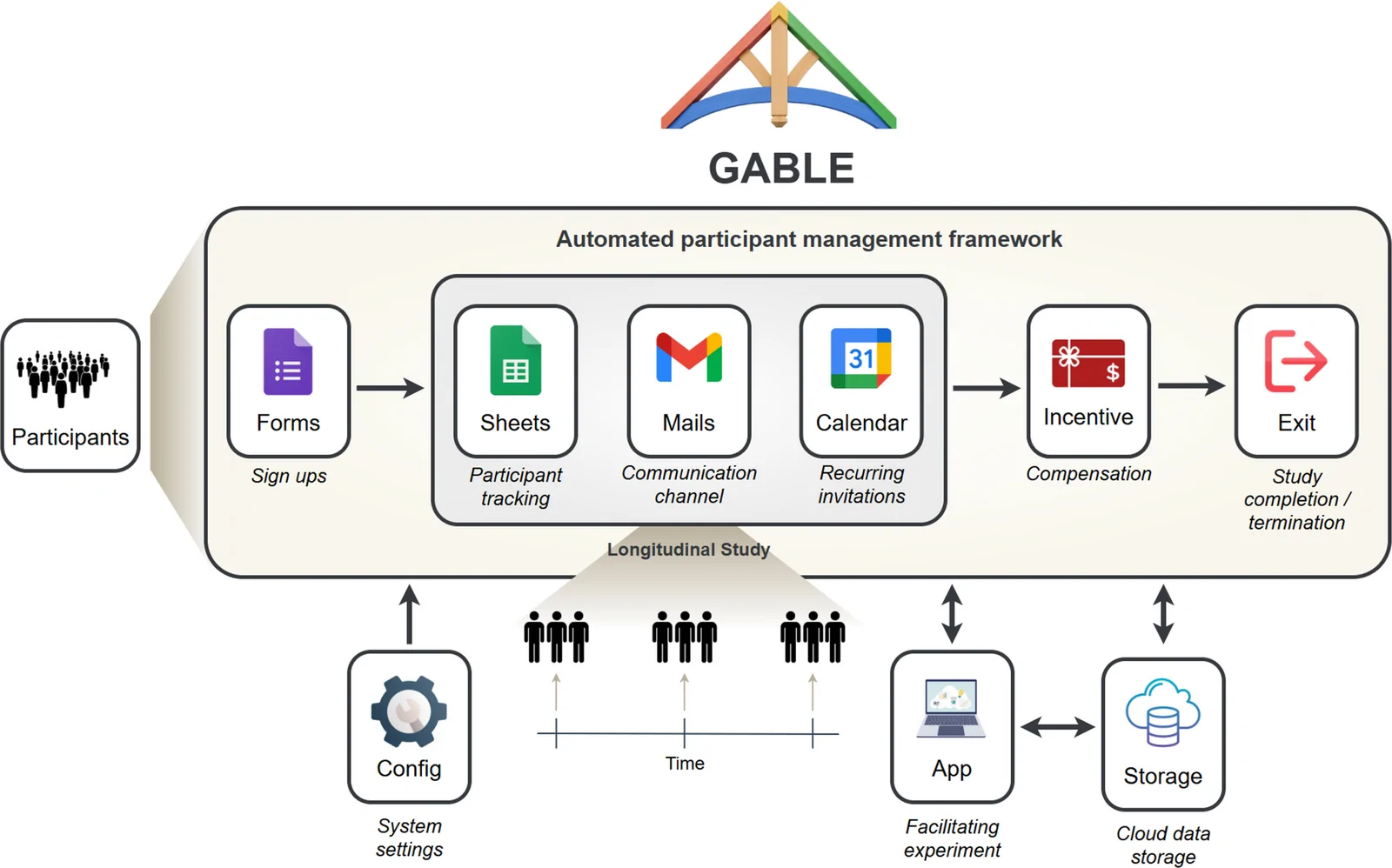
GABLE architecture illustrating automated participant management and integration with experimental and data-storage components

The GABLE platform consists of three interconnected layers—participant management, longitudinal task execution, and data integration—that together automate the full life cycle of a longitudinal study. The participant management layer oversees recruitment, scheduling, communication, and compensation using tools such as Forms, Sheets, Gmail, and Calendar. The task execution layer interfaces with external web applications where study activities occur, while the data integration layer keeps participant progress synchronized with the external storage that these applications read. Configurable study parameters, including timing rules, message templates, and incentive policies, govern all automated operations, allowing researchers to maintain centralized control of complex longitudinal workflows. As shown in Fig. 1, GABLE unifies these components into a single, continuous system that links participants’ progress from sign-up to study exit.

Participant-level study metadata, used to manage scheduling and track progress, does not include experimental or behavioral data collected during the experiment itself. All such metadata and the associated automation scripts are stored within the security context of the GABLE user’s Google Workspace account. Access is governed by that user’s existing authentication and sharing controls. Figure 1 shows how GABLE coordinates participant-facing processes with backend progress tracking.

GABLE runs entirely within the Google Apps Script environment, a cloud-based JavaScript platform hosted on Google’s servers and bound to the researcher’s Google account, so no local server, installation, or separate hosting is required. Rather than being provided as a hosted service or external library, GABLE is distributed as source code that researchers copy from its public repository into their own Apps Script project. Because each deployment is the researcher’s own copy, the system can be adapted or extended freely.

A study is configured through a global configuration file, and setup involves copying the scripts into the Apps Script editor, editing that file, and running an initialization routine, after which the study runs automatically and is monitored from the linked tracking sheet. Activity is also recorded in execution logs, which require no additional setup and can be viewed in the Executions panel of the Apps Script editor, where every scheduled or triggered run appears with its status and messages. Full setup and configuration instructions are provided in the GABLE repository (https://github.com/rail-cwru/gable).

**Fig. 2:**
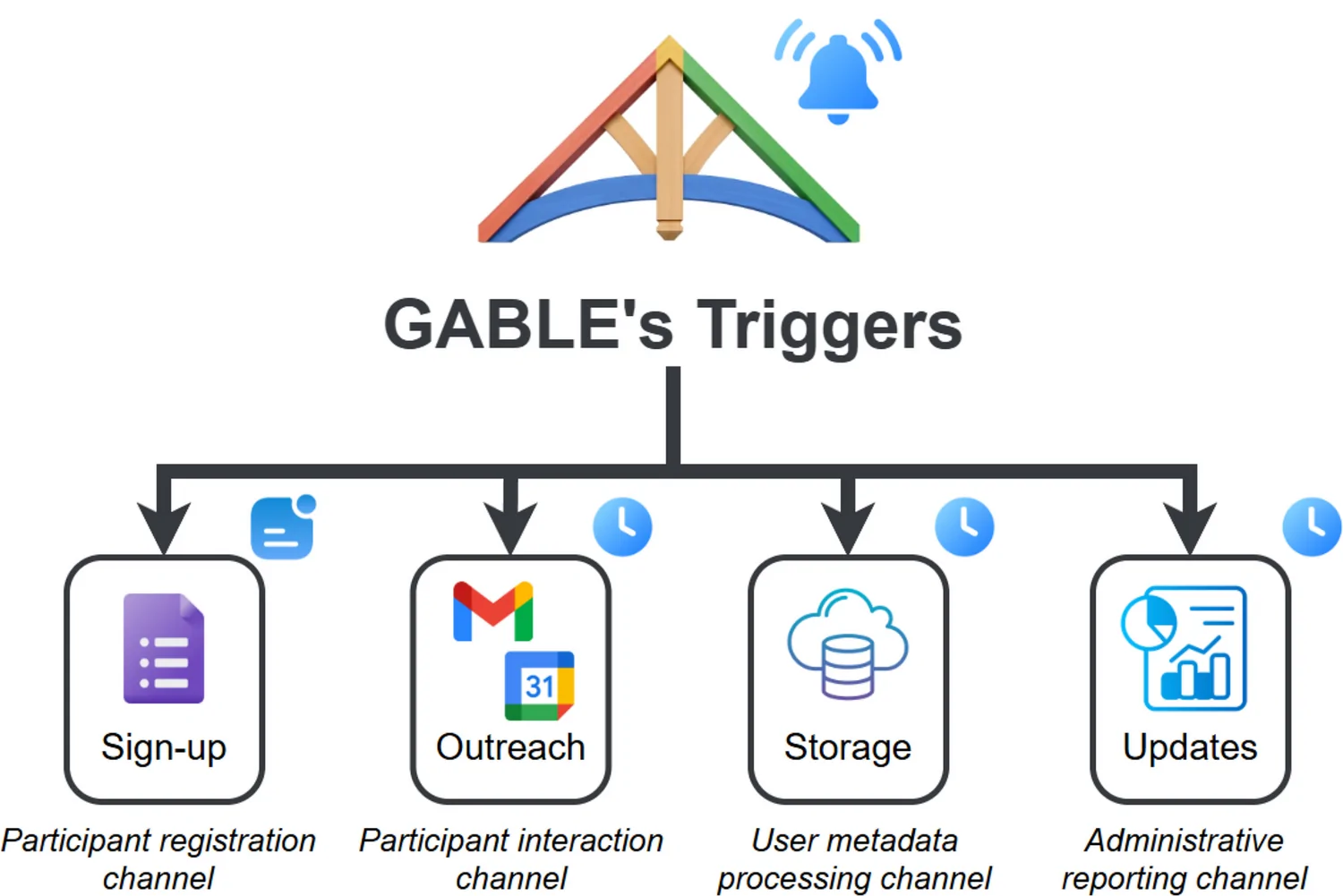
GABLE’s trigger system with event-based and time-based triggers

At the core of GABLE’s automation is its trigger system, which is illustrated in Fig. 2. Sign-up is handled by an event-based trigger that fires when a sign-up registration form via Google Forms is submitted, creating the participant record and initializing study settings. All other channels run on time-based triggers: Outreach runs on scheduled intervals to send invitations and reminders; Storage periodically processes and syncs metadata; and Updates periodically generates administrative summaries and status reports. Together, these routines keep communication, progress tracking, and record-keeping synchronized without manual intervention. The next section details the specific scheduling rules and Apps Script functions behind each trigger.

**Fig. 3:**
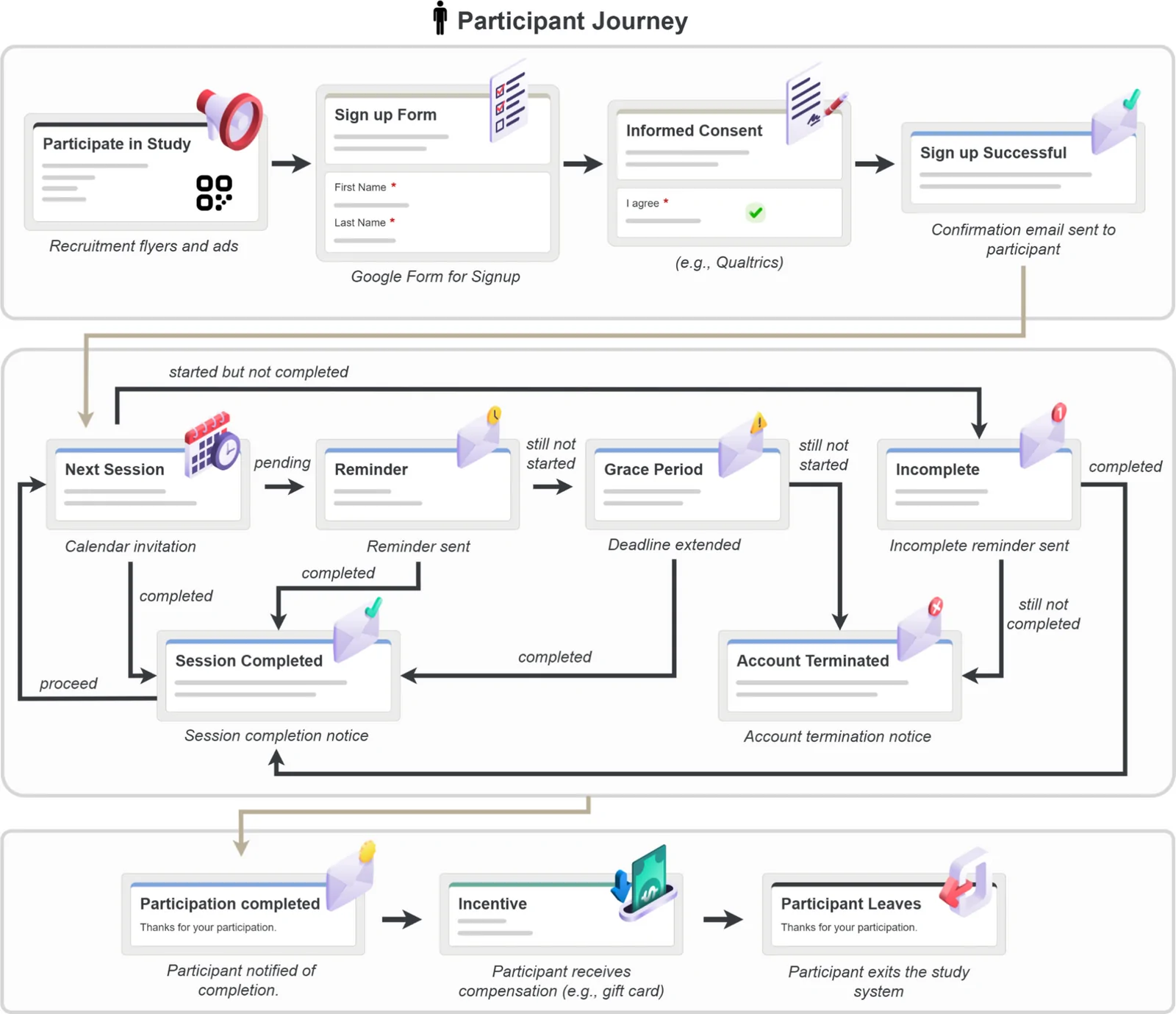
Participant journey in the GABLE platform

Figure 3 summarizes the participant journey in a GABLE-managed study, illustrating how the system operationalizes each component from the user’s perspective. Participants progress through three main stages: (1) recruitment and onboarding via linked study sign-up registration and consent forms, (2) session scheduling and progress management supported by automated reminders, grace periods, and inactivity handling (e.g., calendar invitations, reminder and reactivation e-mails), and (3) study completion with incentive delivery and structured participant exit (e.g., automated gift card e-mails and termination confirmations). Each transition is governed by real-time tracking and personalized communication, ensuring sustained engagement with minimal administrative oversight.

### Participant recruitment and enrollment

To recruit participants through GABLE, researchers distribute recruitment materials (e.g., flyers) with a link to a sign-up registration form designed by the researchers and configured by GABLE’s initialization script, as illustrated in Fig. 3 (top). Because the sign-up registration form is a standard web link, researchers have flexibility in how they recruit, such as flyers or advertising through external recruitment platforms or subject pools. Once a participant completes the sign-up form and provides a contact e-mail, GABLE manages the remainder of the study independently. To this end, GABLE complements existing recruitment channels rather than integrating with or replacing their participant-management features. The form collects essential participant information such as name, institutional e-mail, and preferred start time. Upon participant submission of the registration form, GABLE automatically creates a persistent participant record in a master tracking Google Sheet. Each record receives a unique participant ID and an experiment group code assigned through a configurable round-robin algorithm. The algorithm dynamically balances groups by considering only participants who are currently active or have completed sessions, excluding invalidated or withdrawn entries. By default, it balances assignment across the defined experimental groups, but it can be extended to balance on additional participant attributes, such as gender or age, by defining corresponding subgroups. This approach maintains an even number of participants in different study conditions despite dropouts. Group mapping parameters, such as the number of study conditions, are stored in a configuration file, allowing reproducible and flexible study deployment.

Unlike ad hoc study setups that require manual transfers or coordination across several disconnected tools, such as separate forms, participant tracking sheets, e-mail, and calendar applications, GABLE establishes explicit linkages between the registration form (and any linked pre-experiment or consent form), the scheduling sheets, and the sign-up confirmation e-mail through Apps Script automation (Fig. 3, top). The sign-up trigger in the participant management registration channel implements this process, as shown in Fig. 2. By generating, linking, and synchronizing all components at runtime, GABLE directly connects enrollment with scheduling and participant monitoring, described in the following section.

### Scheduling and session management

Once participants are enrolled, GABLE governs their longitudinal progression through an adaptive scheduling system that dynamically regulates session availability, timing, and reminders, as illustrated in Fig. 3 (middle). GABLE automates participant progression through multi-session studies by linking session availability to each individual’s completion history rather than relying on a rigid, pre-scheduled timeline. At the time of enrollment, the system reads parameters, such as the total number of sessions and the allowed interval between them (for example, 1–3 days), from its configuration file. This file centralizes all study settings as editable parameters, including the number of sessions, group definitions, session intervals, and incentive amounts. These parameters define the timing rules that determine when participants may begin their next session. A complete, working configuration is provided in the repository, alongside a fictitious simulated example task, STROBE (Stroop Task for Repeated Observation of Behavioral Effects), a jsPsych-based implementation of the classic Stroop task (Stroop, 1935), built to integrate with GABLE from sign-up through completion. All scheduling and invitation triggers mentioned below are managed through the Participant Interaction channel in the trigger system (Fig. 2).

Next, the system determines the participant’s first session start time based on their preferred time as provided in the sign-up form. The system then generates and sends a corresponding calendar invitation, anchoring the study’s start to the participant’s chosen preferred day. From that point onward, GABLE manages all subsequent sessions dynamically.

When a participant finishes a session, the task application records the new status in the shared participant state file (see Metadata management). GABLE’s time-based trigger reads the file on a fixed schedule to register the completion, then checks whether the required waiting period has elapsed. This shared-state design, rather than a record being detected through a callback or end-redirect URL, keeps GABLE host-agnostic. The task application and GABLE never communicate directly, so GABLE can work with any experiment platform capable of reading and writing the shared file. Once eligible, the next session is unlocked and a new access link is delivered to the participant automatically. If a participant does not begin the session within the allowed interval, the system issues reminder e-mails according to customizable templates and timing rules defined in the configuration file.

For longer studies, researchers can specify when grace periods become active. For instance, in a 20-session study, a grace extension might only apply after the tenth session, allowing greater leniency for participants who have already demonstrated engagement. These options are set as parameters in the global configuration file, including whether grace periods are enabled, the session after which they begin, and how many additional days they allow, with a working example provided in the repository configuration.

If a session is started but not completed within the allocated time, a separate inactivity reminder sequence is triggered, prompting participants to resume before the session expires. This includes participants who leave a started session incomplete beyond an allowed window, or who miss a session deadline after any grace period. These thresholds, including the inactivity window and the grace period, are configurable.

**Fig. 4:**
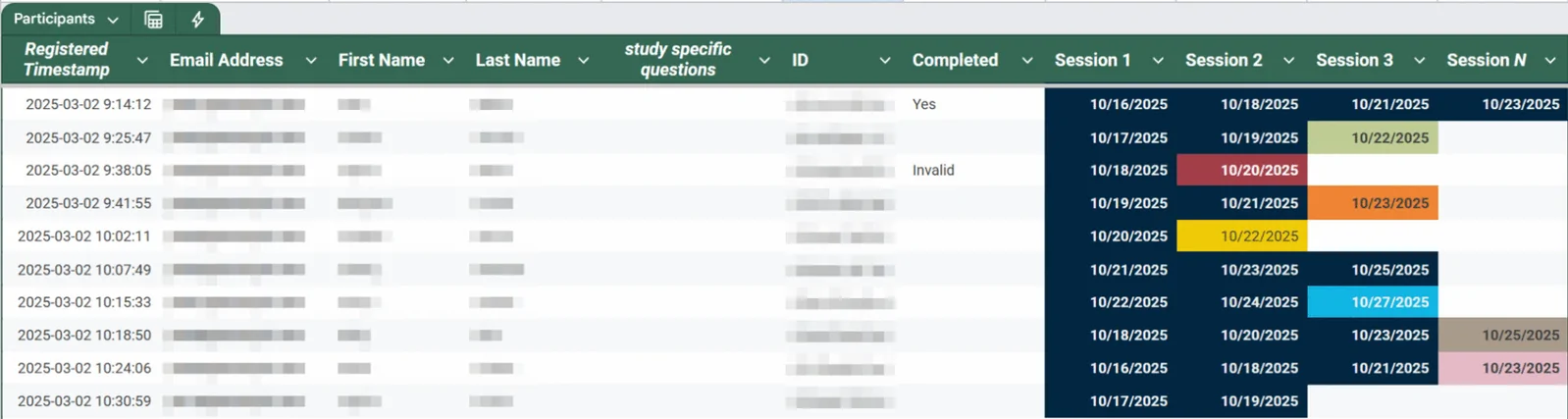
Real-time participant tracking sheet generated by GABLE. *Note.* Rows represent participants and columns represent sessions; cell colors indicate status (e.g., *dark blue* = completed, *green* = upcoming, *red* = invalid)

The centralized tracking sheet functions as the control center of the study, reflecting each participant’s status, including session starts and completions and their timestamps, in real time. Each row represents a participant, and each column corresponds to a session, with cell colors automatically indicating status (an example schema is shown in Fig. 4). For example, green denotes sessions ready to launch, blue indicates completed sessions, yellow flags approaching deadlines, orange marks partial completions, grey and purple indicate active grace periods, and red signals invalidated participation. The schema is fully customizable; new operations, status rules, or color codes can be added or modified through the configuration file to fit different study requirements, as documented in the repository. Because the sheet is updated automatically, it always reflects the current state of the study and requires no manual upkeep, allowing coordinators to assess progress and identify issues at a glance across hundreds of participants.

### Automated communication pipeline

GABLE’s communication pipeline translates participant status updates into automated, personalized messages. Rather than relying on static reminders or manually scheduled e-mails, the system continuously monitors the master tracking sheet to determine which participants require contact and what type of message they should receive (see Fig. 3, middle). Each communication event, such as an invitation with a study link, reminder, completion notice, or other study e-mail, is triggered by a specific state change in the participant record and is executed through the Participant Interaction channel in the trigger system (Fig. 2).

The pipeline operates through Apps Script functions linked to built-in Gmail and Calendar services (note: participants need *not* have Gmail accounts), with setup handled entirely within the Google Workspace environment rather than through custom code. Message templates are defined in a configuration file that specifies their subject lines, timing rules, and trigger conditions, with example templates and study-calendar settings for the administrators provided in the repository configuration. When the system detects that a condition has been met (for example, a new participant enrollment, a session becoming available, or a deadline approaching), it generates the appropriate message, substitutes participant-specific fields such as ID, name, and session number, and delivers the e-mail automatically.

In addition to communications sent to participants’ preferred e-mail addresses, GABLE issues calendar invitations through its script-driven communication workflow. These invitations serve as an additional reminder layer, placing upcoming sessions directly on the participant’s calendar (e.g., Google Calendar, Outlook) without requiring manual setup. Invitations are created as structured attachments within the e-mail process and can be refreshed or resent if a session’s timing changes. To keep the research administrators’ own calendars organized, GABLE allows the use of a dedicated study calendar within the same account, so all automated invitations appear under a separate calendar list while still being delivered from the research administrator’s e-mail address.

Each type of message is tied to a corresponding event in the participant lifecycle. The system sends enrollment confirmations and onboarding instructions immediately after registration, followed by calendar invitations for the first session. Once a session is completed, the system issues an acknowledgment and, when applicable, activates the next session invitation. If a participant does not begin or finish a session within the allocated window, reminder messages are sent at predefined intervals, followed by optional grace-period alerts when extended time becomes available. These communications stay synchronized with the scheduling logic so no message is sent out of sequence or outside the participant’s permitted timeline.

All outgoing messages and calendar invitations remain traceable through both the linked e-mail account and the script’s execution logs (see the Platform overview), allowing researchers to confirm when communications were issued without maintaining a separate record. Because the configuration file defines each message’s content, timing, and conditions, researchers can adjust communication behavior without editing the underlying code. This integration ensures that every operational update, whether a reminder, deadline extension, or calendar event, is handled through the same adaptive and unified messaging system.

### Incentive management and delivery

Once a participant’s involvement in the study ends, either through successful completion of all required sessions or through early termination (for example, after repeated missed deadlines), GABLE moves them into a structured exit phase, as illustrated in Fig. 3 (bottom). The system updates their status in the tracking sheet, stops scheduling additional sessions, and sends a final completion or exit e-mail thanking them for participating and, when appropriate, providing debriefing information or next steps. After this communication, the system hands off their record to the incentive module described below, and once any compensation is processed, the participant receives a final notification and formally leaves the study system.

GABLE automates participant compensation by linking verified study progress to a managed inventory of electronic gift cards. Each participant’s earnings are calculated dynamically based on the study’s configured reward structure, for example, a fixed amount delivered per completed session with an additional bonus for full completion. This structure is stored in the configuration file, allowing researchers to define whether compensation is session-based, cumulative, or milestone-triggered. Once a qualifying event occurs, the system initiates the incentive allocation process.

The corresponding gift card code is selected from an available pool stored in a dedicated sheet, ensuring that each code is used once and linked to a specific participant ID. The allocation logic matches owed amounts to available denominations, updates the card’s status to “used,” and records the planned payment for administrative review. Participants receive payment notifications and codes through the same automated communication pipeline used for study reminders and completion e-mails (Fig. 2, participant interaction channel). By connecting progress information, incentive records, and delivery within one workspace, GABLE removes the need for manual tracking and ensures consistent, auditable payouts across all participants.

### Metadata management

GABLE manages all study metadata within Google Workspace and supports an external storage layer for participant state files. Here, study metadata refers to the coordination data used to run the study, such as each participant’s study ID, group assignment, session completion status, scheduling times, and current status, and is distinct from the experimental or behavioral data collected during the task. Participant information is maintained in two coordinated locations: core study records in Google Sheets and an external file containing only the participant ID, group assignment, and scheduling data used by the task delivery application to configure the experiment seen by a participant. This external file can be hosted on any provider such as Azure^6^ or S3,^7^ and GABLE remains compatible with alternative storage services. GABLE keeps identifiable participant records, such as name and e-mail, within Google Workspace, and shares only the participant’s study ID with the task application. The experimental data therefore remains separate from personally identifiable information, and is only linked to GABLE’s records through this participant study ID. Keeping identifiers out of the experimental data also makes it easier to analyze and share, as the dataset can be handled without exposing personal information (assuming the task itself collects no private information).

Participant activity and study metadata are continuously synchronized so that external applications, such as the experiment web interface, always access the most up-to-date participant information. The Apps Script layer maintains synchronization by reading study metadata for all routine operations and writing only at key events such as initializing the file at sign-up, updating the next scheduled session time, and terminating the account. These read–write operations are executed through the Storage channel in the study metadata processing track of the trigger system (Fig. 2).

The participant state file is a small per-participant record (for example, pID_gable.json) that holds the participant’s session and trial progress, including the current session number, completion flags, and timestamps. GABLE creates this file at sign-up, the externally hosted task updates it as the participant works through the study, and GABLE reads it periodically to advance sessions and update the tracking sheet. Researchers using GABLE should ensure that their externally hosted task updates this file. The repository README describes how to do this, and the task-example folder provides a working implementation. This shared store is a consequence of hosting the task externally rather than a constraint imposed by GABLE; its location and provider are left to the researcher, and it need only be reachable over HTTP. The deployment reported here used Azure, but any writable storage can be used, including options with free tiers. This file-based design, in which GABLE polls the store for critical events such as session initiation and completion, is deliberately simple and robust. Because each update is written to durable storage and read on GABLE’s schedule, a transient outage or network delay on either side does not cause an update to be lost, as GABLE simply reads the latest state on its next poll. It could additionally be extended to support a push-based approach, in which the task notifies GABLE directly rather than being polled.

### Administrative oversight

Summaries and reports are generated through the Updates channel in the administrative reporting track of the trigger system (Fig. 2). The Updates channel maintains a dedicated tab in the spreadsheet that accumulates daily counts of key operational events, such as sign-ups, session and study completions, missed or invalidated sessions, reminders sent, grace periods granted, and remaining gift-card stock, and periodically compiles these into a status-summary e-mail to the study coordinators. The Updates channel is documented in the repository. Error handling and diagnostic information are accessible through the built-in Apps Script execution logs, which record the exact point of failure, timestamp, and function name for each event. This transparency allows administrators to identify and address issues quickly, minimizing downtime and ensuring smooth study operation.

Most day-to-day administrative tasks can be handled directly within the linked tracking sheet without requiring any code modification. Researchers can update session statuses, extend deadlines, or modify study parameters simply by editing predefined fields in this sheet. For example, if a participant has started a session but not yet finished it, a coordinator can extend the window for completing that session directly in the sheet. Similarly, if a participant reaches out to request more time, for instance because of travel or illness, a coordinator can grant an additional day or shift their next session date directly in the sheet. The automation layer detects and propagates these changes across connected components, ensuring consistency throughout the system. This spreadsheet-based control allows immediate intervention when needed while preserving the underlying automation. Because these operations are performed by editing spreadsheet fields rather than writing code, they require no programming, and the shared sheet supports collaborative study management.

For advanced intervention or batch operations, GABLE also offers a lightweight administrative scripting interface. Through external API calls or Python scripts, users can execute advanced GABLE functions, such as reactivating or rescheduling a participant, regenerating e-mail reminders, or triggering a study-email pass, each with a single call, without halting the live study. The repository documents an example call and the available operations. These scripts interact safely with the automation layer and use standardized authentication to protect study integrity. This hybrid architecture, blending full automation with human oversight, lets researchers maintain flexibility and precision while keeping the system stable, scalable, and transparent throughout extended deployments.

## Methods

We demonstrate GABLE’s capabilities through deployment in two ongoing real-world longitudinal studies on cognitive skill acquisition (to be reported elsewhere; not preregistered).

### Participants

A total of 852 participants were recruited across the two studies, completing browser-based cognitive tasks and self-report measures. Of all the initial enrollments, 593 participants completed all 20 sessions, while 623 participants completed at least the first ten sessions.

### GABLE system and the procedure of studies

Both studies require each participant to complete 20 sessions scheduled approximately every 2 days, with each session becoming available 24 h after the previous completion and remaining open for a 1–3-day window. The experimental tasks are delivered through custom-developed web applications, one built in Unity and exported to WebGL and the other built with Node.js. These components handle task delivery and are therefore external to GABLE.

GABLE automates the entire experimental workflow with minimal manual oversight. The system manages session scheduling, enforces protocol timing constraints, triggers personalized e-mail reminders, tracks participant progress, and handles compliance violations without researcher intervention. The system automatically invalidates participants who miss session deadlines or become inactive, preserving protocol integrity. To support participant retention, we implement a grace period policy for participants who complete at least ten sessions (half the protocol), extending their completion window by one additional day and sending targeted reminder communications.

For data management in these deployments, we integrated Microsoft Azure as the cloud storage solution for experiment data and system logs. However, GABLE’s architecture is storage-agnostic and can interface with any cloud provider or database system. This flexibility, combined with the Apps Script-based automation layer, enabled us to support two large-scale longitudinal studies with minimal technical expertise or server maintenance, requiring no dedicated servers, database administration, or specialized programming skills, and no infrastructure costs for the participant management system itself.

## Results

As of this writing, GABLE has processed 12,982 session completions. Of all the initial enrollments, 593 participants completed all 20 sessions, while 623 participants completed at least the first ten sessions. The system automatically invalidated 604 accounts due to missed deadlines or inactivity, demonstrating its capacity to enforce protocol compliance without manual monitoring. GABLE sent 5812 standard reminder e-mails and 570 grace period notifications, with 39 requiring grace period-specific reminders. Research staff needed manual intervention only for occasional participant requests, such as deferring study start dates or addressing technical inquiries.

One may wonder if GABLE influenced the dropout rate for these studies. Because the studies did not include a non-GABLE comparison condition, we cannot attribute the observed retention to the system itself. A direct comparison of the observed attrition rate with other studies is difficult, because the protocols are distinctive in their tasks, number of sessions, and timing intervals. The observed dropout is nonetheless consistent with the demands of an intensive, multi-session, multi-week design, in which sustained participation across 20 sessions is inherently challenging. GABLE’s automation is unlikely to have changed these underlying drivers of retention; its role is to deliver scheduling and reminder communications reliably and on time, which may have helped support continued participation.

During these deployments, GABLE demonstrated high operational reliability with no participant-facing failures. Over a representative 7-day period, the system executed approximately 1900 automated operations with a total logged error rate of 2.30%. These executions covered all major processes, including participant state updates (e.g., session timing, reminder scheduling, grace-period activation), e-mail delivery, and new sign-up registrations. Most recorded errors reflected transient API timeouts or network delays within Google’s execution environment and were automatically resolved through redundant trigger routines. The email-update function, which distributes periodic study-status summaries to coordinators, and the registration function, which initializes participant records upon sign-up, both maintained a 0% failure rate. The time-based triggers that read the Azure metadata and distributed study e-mails ran every 5 min and hourly, respectively, with error rates of 2.47% and 0.34%. These cadences are configurable and were the values chosen for these deployments rather than an upper bound; Apps Script time-based triggers can run as often as once per minute, allowing shorter intervals when a study requires tighter timing. In practice, GABLE’s timing precision is governed by the configured trigger cadences, and in these deployments the triggers executed reliably and close to their scheduled times. Participant progress was read every 5 min, so session completions were detected promptly, and study e-mails were dispatched hourly, a cadence well matched to a protocol in which sessions open approximately 24 h apart. Because these cadences are configurable down to once per minute, a study requiring tighter timing can simply increase the trigger frequency, delivering communications within minutes of an event. Throughout these deployments, this timing performed reliably, with all events delivered within their intended windows.

Because critical operations are supported by multiple redundant triggers, minor interruptions had no effect on study progression or participant experience. Manual intervention was only required for one-off administrative actions such as a participant requesting a changed start date before beginning their study.

## Discussion

This work highlights how lightweight infrastructure, built entirely with off-the-shelf tools, can support fully automated longitudinal research at scale. While much of the focus in online experimentation has centered on task design and delivery, GABLE addresses critical yet often overlooked layers of the workflow: Participant coordination, scheduling, session tracking, and protocol adherence across extended timelines. By emphasizing automation and operational logistics, GABLE offers a pragmatic model for running complex, multi-session studies.

GABLE’s deployment in two live longitudinal studies shows that scalable participant management and automated scheduling can be achieved using only standard cloud-based tools. Across thousands of automated operations, the system maintained stability and protocol fidelity without requiring continuous oversight, indicating that much of the administrative workload typically handled by research staff can be reliably transferred to automation. The framework’s ability to coordinate hundreds of participants, enforce timing rules, and manage individualized communication at negligible cost highlights the practical feasibility of low-infrastructure approaches to longitudinal experimentation. More broadly, these results suggest that much of the technical barrier to participant management in multi-session studies may stem less from their inherent design than from the absence of accessible automation tools.

GABLE integrates with existing experimental tools and data services, positioning itself as an automation layer rather than a replacement for established task builders or recruitment platforms. As an example, this integration model is demonstrated by the jsPsych-based example task provided in the repository, which connects to GABLE from sign-up through completion. The server and cloud-storage components used in this example reflect the setup of our studies rather than requirements of GABLE; the framework only needs the task to record its progress to a writable location it can read, so lighter or free storage options can be used instead. This interoperability allows researchers to retain their preferred experiment hosts and frameworks, such as Gorilla, jsPsych, or any custom developed web-based task application, while offloading scheduling, reminders, and progress tracking to GABLE.

Unlike purpose-built commercial systems or institutionally hosted platforms, GABLE requires no custom infrastructure. Built entirely on Google Apps Script and other native Google Workspace tools (Forms, Sheets, Calendar, Drive), the platform incurs no additional expenses for research and noncommercial use beyond a standard Google account. This makes GABLE highly accessible to individual researchers, graduate students, or labs operating without dedicated funding for technical infrastructure. GABLE also offers low-friction deployment. Although adopting GABLE requires learning how it works, working with it is largely a matter of configuration through familiar Google Workspace tools such as Forms and Sheets, rather than learning a general-purpose programming framework, managing cloud servers, or writing backend code. The setup steps are described in the Platform Overview, and the system can be adapted to a range of study designs, making it well suited to rapid prototyping and to teams without dedicated technical infrastructure. Finally, GABLE is scalable within reason. While it is not designed to handle millions of participants, it is well-suited for studies involving hundreds to thousands of participants across dozens of sessions. The system’s modular structure and use of cloud-native services allow it to handle participant tracking, reminder scheduling, and data management reliably over extended periods.

## Limitations

While GABLE lowers many barriers to running longitudinal studies, it is not without trade-offs. Despite its accessibility, GABLE’s reliance on Google Workspace may introduce practical and institutional constraints. Many universities and clinical institutions maintain data-governance policies that restrict the use of third-party cloud services, particularly when operated through personal or non-institutional accounts. Although GABLE can be deployed within institutional Google domains to satisfy compliance and data-ownership requirements, such configurations depend on local IT infrastructure and administrative policies rather than on the framework itself. These considerations emphasize the need to align automation tools with institutional privacy standards and review procedures.

GABLE’s functionality is also inherently tied to the Google ecosystem. The system integrates with Workspace tools such as Forms, Sheets, Gmail, and Calendar, which may not be supported in environments that rely primarily on other infrastructures, such as Microsoft 365 or locally managed servers. This dependence may limit adoption with institutional accounts in such cases. Alternative platforms (such as using Microsoft Power Automate, Outlook, and SharePoint) could achieve similar functionality, though it would require a distinct architecture and additional configuration effort.

The system also inherits the performance limits and execution quotas of the Google Apps Script environment, which may affect extremely high-volume studies. In practice, occasional throttling or transient API timeouts were rare and automatically recovered through redundant triggers, with no participant-facing disruptions, and we did not encounter any Apps Script or Gmail quota limits during these deployments. Such quotas do exist and could bound very large studies; the most relevant is the daily cap on the number of e-mail recipients that Apps Script applies to outgoing messages, which scales directly with participant communications. This cap is substantially higher on institutional Google Workspace accounts (on the order of 1500 recipients per day) than on personal accounts, so studies approaching these volumes are best run on a Workspace account. These constraints do not undermine the framework’s stability but highlight the importance of understanding execution quotas and designing study schedules accordingly when scaling to larger projects.

Relatedly, GABLE’s timing precision is bounded by its trigger cadence rather than by real-time execution. Because periodic triggers drive scheduling and communication, time-sensitive events are acted upon within the configured interval rather than instantaneously. This makes GABLE well suited to studies whose session timing tolerances are on the order of minutes to roughly an hour, such as sessions that open a day apart, but less suited to paradigms that require sub-minute or exact-instant session timing. Where tighter timing is needed, increasing the trigger frequency narrows this window, at the cost of more frequent execution.

Finally, like any cloud-based system, GABLE’s overall reliability could be influenced by the stability of network connections, such as the experiment web pages, web servers, database connections (i.e., APIs), or third-party cloud storage services. While these components operate independently of GABLE’s automation logic, intermittent delays or connection failures can temporarily affect synchronization or data updates. Such dependencies are inherent to distributed research architectures and should be considered when integrating external components into the system.

## Conclusion

We have presented GABLE, a Google Workspace-based system that automates participant scheduling, reminders, progress tracking, and incentive delivery, addressing a critical yet overlooked challenge in longitudinal research: coordinating hundreds of participants across multiple sessions without custom infrastructure. Table 1 shows GABLE’s advantages over existing platforms. The development and deployment of GABLE show that longitudinal studies can be effectively managed using standard cloud-based tools without specialized infrastructure. By establishing automated communication channels and coordinating session management using tools already familiar to many researchers, GABLE lowers the barrier to running complex, multi-session studies. Its successful use in two large-scale cognitive psychology studies demonstrates stable performance, reliable timing control, and minimal researcher oversight. These results suggest that accessible automation can enhance the feasibility and reproducibility of longitudinal research across a wide range of experimental settings.

## Data Availability

Materials, example code, and the software implementation used in this study are available at https://github.com/rail-cwru/gable.

## Footnotes

1 Qualtrics, https://www.qualtrics.com

2 Gorilla, https://gorilla.sc

3 E-Prime Go (Psychology Software Tools), https://pstnet.com/products/e-prime-go

4 These are examples of third-party storage and data-collection services: Qualtrics (https://www.qualtrics.com), Box (https://www.box.com), REDCap (REDCap Software, n.d.), and the Open Science Framework (OSF; https://osf.io).

5 https://github.com/rail-cwru/gable

6 Microsoft Azure Blob Storage, https://azure.microsoft.com

7 Amazon Simple Storage Service, https://aws.amazon.com/s3

## Code availability

The code used in the present study (GABLE) is available at https://github.com/rail-cwru/gable.

## Declarations

### Open practices statement

The source code for GABLE is publicly available for noncommercial research use at https://github.com/rail-cwru/gable. The manuscript describes a software infrastructure; no participant-level datasets are released as part of this article. The illustrative case studies referenced in the manuscript were not preregistered.
